# The association between altitude and serum folate levels in Tibetan adults on the Tibetan plateau

**DOI:** 10.1038/s41598-022-22968-6

**Published:** 2022-10-25

**Authors:** Shaoli Yao, Xiwen Chen, Yao Zhou, Li Xu, Qi Zhang, Shimin Bao, Huiru Feng, Weihong Ge

**Affiliations:** 1 Department of Neurology, Hospital of Chengdu Office of People’s Government of Tibet Autonomous Region, Chengdu, Sichuan China; 2 Department of Neurology, The Second Affiliated Hospital of Chengdu Medical College, China National Nuclear Corporation 416 Hospital, Chengdu, Sichuan China

**Keywords:** Health care, Medical research, Risk factors

## Abstract

This study investigated the relationship between residence altitude and serum folate levels in healthy Tibetans living on the Tibetan Plateau. Participants were selected from those who underwent physical examinations at our health center between November 2019 and February 2020. Demographic characteristics and medical histories were collected, and fasting blood was tested for serum folate and other hematological indicators. The relationship between altitude and serum folate levels was analyzed using a multivariable linear regression model. Serum folate levels were associated with altitude (*β* = − 0.44; 95% confidence interval [CI] − 0.71; − 0.16), hemoglobin (*β* = − 0.01; 95% CI − 0.03; − 0.00), red blood cells (*β* = − 0.72; 95% CI − 1.18; − 0.27), hematocrit (*β* = − 0.07; 95% CI − 0.12; − 0.02), high-density lipoprotein cholesterol (*β* = 2.67; 95% CI 1.35; 3.98), and sex (*β* = 0.68; 95% CI 0.12; 1.23). Multivariate linear regression analysis revealed that altitude was negatively associated with serum folate levels. After adjusting for confounding factors, serum folate levels decreased by 0.33 ng/mL per each 500-m increase in altitude (*β* = − 0.33; 95% CI − 0.6; − 0.05; *P* = 0.022). Altitude was negatively associated with serum folate levels in Tibetan adults. The relationship between altitude and folate levels should be further explored in populations of different races and disease states. Further large-scale prospective studies should illustrate the causality of this relationship.

## Introduction

Folate is a water-soluble B-vitamin that plays an important role in maintaining human health. The reported prevalence of folate deficiency varies considerably worldwide due to the different definitions of low serum folate levels, and the population studied. The prevalence of folate deficiency has been reported to be less than 3.2% in children^1–3^, 46%, and 3–12% in pregnant or lactating women^4^, respectively, and less than 4% in the healthy population^5,6^. In a nutritional survey, Gupta et al. discovered a 1.5% prevalence of folate deficiency in schoolchildren aged 6–18 years at high altitudes in Himachal Pradesh^7^, while Osei et al. discovered low folic acid concentrations in 67.9% of schoolchildren in Himalayan villages in India^8^.

Folate deficiency is associated with several chronic diseases. Several studies have shown that folate deficiency is associated with decreased cognitive function and that folate supplementation may improve cognitive performance^9–12^. A systematic review suggested that folate deficiency is associated with an increased overall cancer risk^13^. Du et al. discovered that low folate concentrations (< 10.5 nmol/L) were substantially linked to a high risk of prehypertension and hypertension^14^. Another study found that folate deficiency was associated with higher serum alanine aminotransferase (ALT) and gamma-glutamyl transferase levels in a Chinese hypertensive population^15^. Furthermore, there is convincing evidence that blood folate levels are closely associated with congenital disabilities. Several studies have identified that low serum folate levels increase the risk of neural tube defects^5,16,17^.

At an average height of 4000 m above sea level, the Tibetan Plateau experiences 30% more ultraviolet (UV) radiation^18^ and a 40% decrease in oxygen content than at sea level^19^. Because of their unique geographical environment, high-altitude areas pose a great challenge to the health of people living there. Prolonged stays at high altitudes have been reported to increase the risk of thrombosis^20^, and a randomized controlled trial in Indian soldiers showed that supplementation with B vitamins such as folate and vitamin B_12_ reduced the rate of thrombosis during a 2-year stay at high altitude^21^. A study by Liu et al. found that ischemic stroke occurs earlier and is more severe in the highlands of China^22^, and an Indian study found that Indian soldiers had a 30-fold higher risk of stroke during prolonged stays at high altitude^23^. A study of the effects of high altitude on pregnancy and newborns found that pregnancy at high altitude was associated with higher rates of congenital malformations, younger than gestational age, and pre-eclampsia^24^.

There are very few studies on the effect of altitude on folate concentrations, and the findings are inconsistent. A randomized controlled trial in Indian soldiers found a significant decrease in serum folate and vitamin B_12_ levels after 1 year at high altitude in a control group (without additional B vitamin supplementation)^21^. In contrast, a study on the effect of high altitude on erythropoiesis found no significant change in intraerythrocytic folate levels after 3 weeks of exposure to an altitude of 6542 m^25^. However, the Tibetan Plateau has an average elevation of approximately 4000 m above sea level^18^, and there have been no studies on the quantitative relationship between altitude and folate levels in healthy populations in the Tibetan areas. Therefore, this study aimed to investigate the relationship between altitude and serum folate levels in a healthy Tibetan population living in the Tibetan Plateau.

## Materials and methods

### Study population

**Figure 1:**
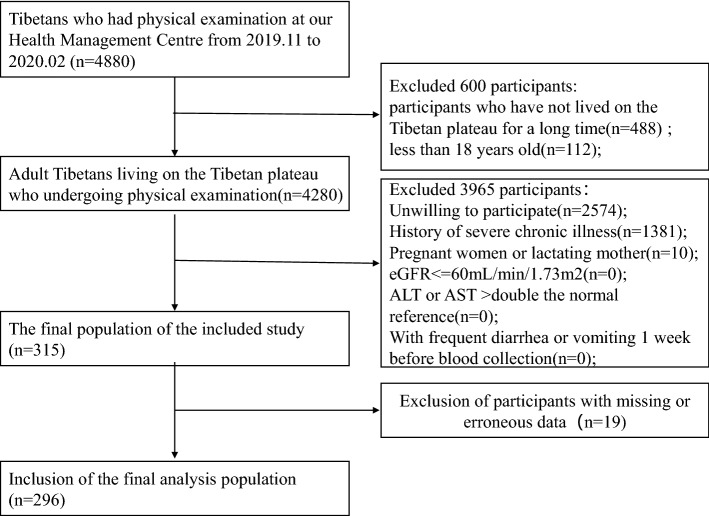
Flowchart of the study. eGFR, estimated glomerular filtration rate.

This cross-sectional study was conducted between November 2019 and February 2020 at the Department of Health Management Centre of the Hospital of Chengdu Office of the People’s Government of the Tibetan Autonomous Region. Finally, 296 consecutive general adults (aged ≥ 18 years) were enrolled among Tibetans undergoing medical examinations at the Health Management Center. The inclusion criteria were as follows: age above 18 years, birth and living on the Tibetan Plateau, spending more than 240 days a year on the plateau, free from severe chronic illness, and willingness to participate. Exclusion criteria were abnormal renal and liver function, taking multivitamin supplements or anticonvulsants, frequent diarrhea or vomiting 1 week before blood collection, untreated intestinal paraspinal diseases or inflammatory bowel diseases, ALT or aspartate aminotransferase (AST) more than double the normal reference, estimation of glomerular filtration rate ≤ 60 mL/min/1.73 m^2^, pregnancy, lactation, and missing or erroneous information on covariates. A detailed flow chart of participant recruitment is shown in Fig. 1. The Hospital of Chengdu Office of the People’s Government of the Tibetan Autonomous Region Ethics Committee (no. [2019]47) approved this study. Signed informed consent was obtained from all participants at study enrollment. The Strengthening the Reporting of Observational Studies in Epidemiology guidelines were used to report this observational study^26^. Statement all methods were performed in accordance with the Declaration of Helsinki and all relevant guidelines and regulations.

### General data collection and diagnostic criteria

Data were collected using a questionnaire. The questionnaire included general demographic information (such as sex, age, height, weight, smoking history, and drinking history), altitude of residence, educational level, occupation, history of illness, recent drug use history, and Mini-Mental State Examination (MMSE) scores. The participants were divided into two groups according to their average age. The smoking status was determined by “Current or former smoker,” and the alcohol consumption was assessed by “Current or former alcohol drinker.”

### Blood sample collection and laboratory measurements

Blood samples were collected from participants who fasted overnight, and blood biochemical and hematological parameters were analyzed in the hospital within 1 h, while a part of the blood sample was rested for 30 min. Then, centrifuged aliquots of serum samples were stored at − 80 °C until analysis for measuring folate levels. Serum folate levels were detected using a Siemens Automatic Electrochemiluminescence Immunoassay (IMMULITE 2000XPI). The intra-assay coefficient of variation for folate levels was 7.8%. The inter-assay coefficient of variation for folate levels was 5.4%. Hematological parameters, including red blood cells (RBC), hematocrit (HCT), mean corpuscular volume, mean corpuscular hemoglobin, and hemoglobin (HGB), were detected by automated flow cytometry. Liver function, including ALT, AST, albumin, and lipid parameters, including total cholesterol, triglyceride, high-density lipoprotein cholesterol (HDL-C), low-density lipoprotein cholesterol, and fasting blood glucose, were measured using an automatic biochemical analyzer (HITACHI 7180).

### Statistical analysis

The distribution of the baseline data of the individuals included in this study is presented for different serum folate groups. Normally distributed continuous variables are presented as the mean ± standard deviation, while non-normally distributed data are presented as the median (interquartile range [IQR]). The normality of the distribution was confirmed using the Shapiro–Wilk test. Categorical data are presented as numbers (percentages). Differences in continuous variables (including body mass index, altitude, age, MMSE scores, and laboratory test results) were tested using the analysis of variance test or rank-sum test, as appropriate. For categorical variables (including sex, education, occupation, smoking, and alcohol consumption), the χ^2^ test or Fisher’s exact test was performed as appropriate. The relationship between altitude and the beta (β) of serum folate was presented using restricted cubic splines with four knots equally spaced at the 5th, 35th, 65th, and 95th percentiles. Multivariable linear regression analysis (beta [β] and 95% confidence interval [CI]) was performed to assess the independent association between altitude and serum folate levels. We applied three models to the regression analysis. Multivariable models were adjusted as follows: Model 1 was not adjusted; Model 2 was adjusted for age and sex; and Model 3 was adjusted for age, sex, RBC, HGB, HCT, and HDL-C. To verify the stability of the results, altitude was used as the independent variable in the multivariable regression analysis and was divided into three groups (< 3500, 3500–3999, and ≥ 4000 m) and two groups (< 4000 and ≥ 4000 m) to be included in the analysis model. Subgroup analyses were conducted using stratified linear regression models. Interactions across subgroups were tested using the likelihood ratio test. All analyses were performed using the statistical software package R (http://www.R-project.org, The R Foundation) and Free Statistics software version 1.5^27^. A two-tailed test was performed, and *P* < 0.05 was considered statistically significant in our study.

## Results

### Baseline characteristics of the study participants

Between November 2019 and February 2020, 4880 individuals underwent medical examinations at our Health Management Center, 296 of whom were included after screening according to the inclusion and exclusion criteria (Fig. 1). The baseline characteristics of the patients are presented in Table 1. The age range was 18–73 years and the mean age was 43.1 ± 12.2 years, and 48.0% were male. The median folate level (IQR) was 4.9 (3.8, 6.1) ng/mL. There were differences in various covariates between the folate groups, including altitude, HGB, RBC, HCT, sex, HDL-C level, and education (all *P* < 0.05).

**Table 1:** Characteristics of the Tibetan healthy adults (n = 296) included in the study.

Variables	Total (n = 296)	Folate < 4 ng/mL (n = 83)	Folate ≥ 4 ng/mL (n = 213)	*P* value
BMI, kg/m^2^	25.5 ± 4.0	25.3 ± 3.6	25.5 ± 4.1	0.643
Altitude, meters	3739.6 ± 503.5	3853.3 ± 485.2	3695.2 ± 504.7	0.015
Age, years	43.1 ± 12.2	42.3 ± 14.0	43.3 ± 11.4	0.519
HGB, g/L	154.5 ± 23.0	161.2 ± 24.1	151.8 ± 22.1	0.002
RBC, × 10^12^/L	5.0 ± 0.6	5.3 ± 0.6	5.0 ± 0.6	< 0.001
HCT, %	45.7 ± 5.8	47.7 ± 5.9	45.0 ± 5.5	< 0.001
MCV, fL	91.5 ± 12.0	90.7 ± 6.4	91.9 ± 13.5	0.448
MCH, pg	30.6 ± 2.8	30.6 ± 2.8	30.6 ± 2.7	0.958
Albumin, g/L	43.8 ± 4.0	43.8 ± 3.2	43.8 ± 4.3	0.956
Glucose, mmol/L	4.9 ± 1.1	4.8 ± 0.8	4.9 ± 1.2	0.683
Cholesterol, mmol/L	4.6 ± 0.9	4.5 ± 0.9	4.7 ± 0.9	0.354
TG, mmol/L	1.3 ± 0.6	1.2 ± 0.6	1.3 ± 0.5	0.749
LDL-C, mmol/L	3.0 ± 0.7	2.9 ± 0.8	3.0 ± 0.7	0.433
HDL-C, mmol/L	1.1 ± 0.2	1.0 ± 0.2	1.1 ± 0.2	0.036
ALT, U/L	28.0 (20.0, 43.2)	32.0 (20.5, 48.0)	26.0 (20.0, 42.0)	0.129
AST, U/L	21.0 (16.0, 27.0)	21.0 (17.0, 26.0)	21.0 (16.0, 27.0)	0.666
MMSE score	25.0 (22.0, 27.0)	25.0 (20.0, 27.0)	25.0 (22.0, 27.0)	0.404
Sex, n (%)				< 0.001
Male	142 (48.0)	56 (67.5)	86 (40.4)	
Female	154 (52.0)	27 (32.5)	127 (59.6)	
Education, n (%)				0.014
Primary or less	44 (14.9)	21 (25.3)	23 (10.8)	
Secondary	30 (10.1)	7 (8.4)	23 (10.8)	
Intermediate	87 (29.4)	19 (22.9)	68 (31.9)	
University	135 (45.6)	36 (43.4)	99 (46.5)	
Occupation, n (%)				1
Farmer and herdsman	17 (5.7)	5 (6)	12 (5.6)	
Other jobs	279 (94.3)	78 (94)	201 (94.4)	
Smoke, n (%)				0.092
No	220 (74.3)	56 (67.5)	164 (77)	
Yes	76 (25.7)	27 (32.5)	49 (23)	
Alcohol, n (%)				0.207
No	160 (54.1)	40 (48.2)	120 (56.3)	
Yes	136 (45.9)	43 (51.8)	93 (43.7)	

Data are presented as the mean (SD) or median (IQR: Q1–Q3) for continuous variables and the percentage for categorical variables.

*BMI* body mass index, *HGB* hemoglobin, *RBC* red blood cell, *HCT* hematocrit, *MCV* mean corpuscular volume, *MCH* mean corpuscular hemoglobin, *TG* triglycerides, *LDL-C* low-density lipoprotein cholesterol, *HDL-C* high-density lipoprotein cholesterol, *AST* aspartate aminotransferase, *ALT* alanine aminotransferase, *MMSE* Mini-Mental State Examination, *SD* standard deviation.

### Univariable and multivariable analyses

Univariate analyses revealed that serum folate levels were associated with altitude, HGB, RBC, HCT, HDL-C, and female sex (Supplementary Table S1). These variables were entered into a multivariate model (Table 2). Multivariable-adjusted restricted cubic spline analyses suggested negative associations of altitude with serum folate (*p* for non-linear = 0.415, Supplementary Fig S1). In the regression analysis, a negative association between altitude and serum folate levels was observed for both altitudes as continuous (altitude scaled to 500 m increments) and categorical variables. After adjusting for confounding factors, serum folate levels decreased by 0.33 ng/mL per each 500-m increase in altitude (*β* = − 0.33; 95% CI − 0.6; − 0.05; *P* = 0.022). When altitude was treated as a categorical variable, the regression analysis showed a gradual decrease in serum folate concentrations with increasing altitude in both the trichotomous (*P* for trend = 0.029) and dichotomous groups. Compared with the group with altitude < 3500 m, serum folate level decreased by 0.25 ng/mL when the altitude between 3500 and 3999 m (*β* = − 0.25; 95% CI − 0.96; 0.46; *P* = 0.490) and by 0.86 ng/mL when the altitude was above 4000 m (*β* = − 0.86; 95% CI − 1.65; − 0.07; *P* = 0.035). Compared with the group with altitude < 4000 m, serum folate levels decreased by 0.68 ng/mL when the altitude was > 4000 m (*β* = − 0.68; 95% CI − 1.31 to − 0.05; *P* = 0.035).

**Table 2:** Multivariable linear regression analyses of altitude associated with serum folate level.

Variable	n	Model 1		Model 2		Model 3	
		*β* (95%; CI)	*P *value	*β* (95%; CI)	*P *value	*β* (95%; CI)	*P *value
**Altitude per 500 m**	296	− 0.44 (− 0.71; − 0.16)	0.002	− 0.39 (− 0.66; − 0.11)	0.007	− 0.33 (− 0.6; − 0.05)	0.022
**Altitude tertials (m)**							
< 3500	60	0 (Ref)		0 (Ref)		0 (Ref)	
3500–3999	150	− 0.36 (− 1.07; 0.35)	0.322	− 0.39 (− 1.11; 0.32)	0.279	− 0.25 (− 0.96; 0.46)	0.490
≥ 4000	86	− 1.15 (− 1.93; − 0.36)	0.004	− 1.01 (− 1.8; − 0.21)	0.014	− 0.86 (− 1.65; − 0.07)	0.035
Trend test			0.003		0.012		0.029
**Altitude in two groups (m)**							
< 4000	210	0 (Ref)		0 (Ref)		0 (Ref)	
≥ 4000	86	− 0.93 (− 1.54; − 0.33)	0.003	− 0.75 (− 1.38; − 0.11)	0.022	− 0.68 (− 1.31; − 0.05)	0.035

Model 1: non-adjusted.

Model 2: adjusted for age and sex.

Model 3: adjusted for age, sex, HGB, RBC, HCT, and HDL-C.

*CI* confidence interval, *Ref* reference.

### Sensitivity analysis

**Figure 2:**
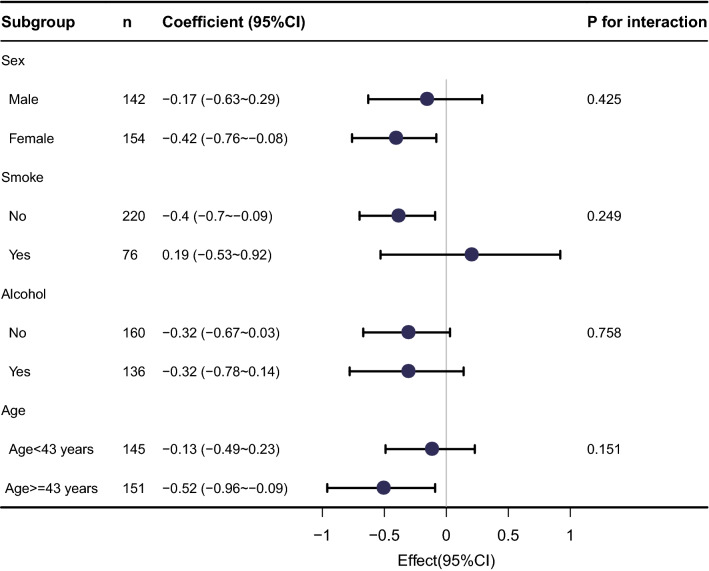
Subgroup analyses of the association between altitude and serum folate level.

Stratification and interaction analyses were further performed to explore whether the negative association between altitude and serum folate levels was confounded by sex, age, smoking, and alcohol (Fig. 2). Folate levels were negatively associated with altitude in both males (*β* = − 0.17; 95% CI − 0.63; 0.29) and females (*β* = − 0.42; 95% CI − 0.76; − 0.08), alcohol drinkers (*β* = − 0.32; 95% CI − 0.78; 0.14) and non-drinkers (*β* = − 0.32; 95% CI − 0.67; 0.03), in subjects aged < 43 years (*β* = − 0.13; 95% CI − 0.49; 0.23) and ≥ 43 years (*β* = − 0.52; 95% CI − 0.96; − 0.09). A positive association was observed only in the smoker group (*β* = 0.19; 95% CI − 0.53; 0.92). Interaction analysis revealed that sex, age, smoking, and alcohol consumption did not significantly interfere with the negative association between altitude and serum folate levels (all *P* > 0.05).

## Discussion

In this cross-sectional study, the folate median (IQR) was 4.9 (3.8, 6.1) ng/mL, which was in accordance with previous studies conducted on Indian students and Iranian adults^6,28^ but less than general people in Beijing, China^29^. In the present study, altitude was independently associated with serum folate levels in Tibetan adults. We observed a negative association between altitude and serum folate levels. The association was reliable and independent of confounding factors, and the results remained stable after the multi-model adjustment and subgroup analysis. To the best of our knowledge, the independent association of altitude with serum folate has not been developed in Tibetans on the Tibetan plateau, and our study found that altitude was negatively associated with serum folate level in a healthy highland population.

Previous studies have observed that serum folate levels in subjects at high altitudes are only 44% of those at sea level^30^. Their findings are in accordance with ours. A significant decrease in serum folate levels after 1 year of residence in the plateau was also observed in the migrant population^21^. However, a study by Richalet et al. found no significant changes in intraerythrocytic folate levels after 3 weeks of exposure to extreme altitudes (6542 m). This difference may be attributed to the different measurements, with serum folate values reflecting recent intakes, whereas erythrocyte folate levels appear to reflect folate intake during the 90- to 120-day life span of erythrocytes^31^.

In this study, a negative association between altitude and serum folate level was observed at both altitudes as continuous (altitude scaled to 500 m increments) and categorical variables. We found that serum folate levels decreased by 0.33 ng/mL per 500 m increase in altitude after adjustment for covariates and confounders by multiple models. The mechanism by which altitude affects serum folate levels is unclear, and we speculated that it might be related to the following mechanisms: (1) decreased folate intake. High-altitude areas have unique geographical characteristics, such as high cold, low pressure, lack of oxygen (the oxygen concentration on the Qinghai-Tibet Plateau is 40% lower than the sea level^18^), drought, and variable climate, which makes their vegetation scarce, and it is difficult to obtain fresh vegetables and fruits. Folate is only found in exogenous foods (e.g., fresh vegetables and fruits, soy products) and supplements^32^, so folate intake is reduced in people in high-altitude areas. (2) UV radiation can degrade folate in human blood and skin^33,34^. The intensity of UV radiation at high altitudes is 30% higher than at sea level^18^. UVB (ultraviolet B, 280–315 nm) radiation degrades the major bioform of folate found in blood, 5-methyltetrahydrofolate (5-MTHF), but does not penetrate the dermis, whereas ultraviolet A (UVA; 315–400 nm) radiation penetrates the dermis but does not degrade 5-MTHF^35^. UVA may indirectly degrade blood 5-MTHF through the production of reactive oxygen species (especially singlet oxygen) by photosensitizers^36^. In addition, the increased need for skin folate supplementation due to intense UV radiation may be another pathway leading to circulating folate depletion, as blood folate is needed to replenish lost folate in the skin^37^. A study in China showed regional and seasonal differences in folate levels from north to south^38^, which may also be related to inconsistent UV radiation intensity from region to region and season to season. (3) Increased frequency of the MTHF reductase (MTHFR) locus rs1801133 allele in the Tibetan population^39^. MTHFR is a key enzyme involved in the metabolic pathway of homocysteine and folate^40^. Tibetans have lived on the Tibetan Plateau for thousands of years, and studies on the genetic basis of high-altitude adaptation in Tibetans have identified two naturally selected allele loci, *MTHFR* and *EPAS1*, which are strongly associated with blood-related phenotypes, such as hemoglobin, homocysteine, and folate in Tibetans^39^. The *MTHFR* locus was strongly associated with folate (*b* = − 0.34, *P*_GWAS_ = 6.5 × 10^−27^, where b is the effect size in standard deviation units) in Tibetan populations^39^. Further studies are needed to explore the mechanism by which altitude affects serum folate levels.

In the subgroup analyses, we observed a positive association between altitude and serum folate levels in the smoker group. In contradiction with our study results, McDonald et al.^41^ conducted research on pregnant women, showing that serum folate was lower in smokers, and Mannino et al.^42^ found that tobacco smoke exposure was associated with decreased folate levels. A previous study observed that folate levels decrease even more with smoke exposure as MTHFR activity declines^41^. Our study observed elevated serum folate levels in smokers at high altitudes. The reason for this phenomenon is still unclear, and these associations are worthy of further investigation.

Our study has some limitations. First, as an observational study, the findings on the relationship between altitude and serum folate levels observed in a healthy Tibetan population do not allow for the derivation of causal conclusions. Second, these results are from a healthy population in the Tibetan Plateau of China, and the interpretation of findings in other populations with different demographics may be limited. Third, although we attempted to statistically correct the bias, we could not exclude unmeasured or residual confounding factors that are vital for the relationship between folate levels and altitude, such as differences in dietary habits. Fourth, due to financial constraints, we were unable to identify the frequency of MTHFR locus rs1801133 allele in our cohort, despite our hypothesis that it could be one of the mechanisms through which altitude negatively correlates with serum folate. Further study is required to confirm this hypothesis. Finally, we did not measure intraerythrocytic folate concentration, which is an indicator of the status of folate intake in the body for 2–3 months. In contrast, serum folate mainly responds to recent folate intake^31^, and Intraerythrocytic folate levels could be measured in the future to further validate the relationship between folate and altitude.

Nonetheless, the strength of this study is that, to our knowledge, it is the first study to investigate the relationship between altitude and serum folate levels in a healthy Tibetan population in China. The altitude of our study population fluctuated from 1500 to 5200 m, providing more reference information on the relationship between altitude and serum folate than in previous studies at a fixed altitude. In our study, we used multimodel-adjusted confounders and subgroup analyses to ensure the robustness of the results. In addition, we included altitude as a continuous and categorical variable in the regression model analysis separately to reduce the chance of data analysis and enhance the results’ robustness.

## Conclusions

In summary, this study suggests that altitude is negatively associated with serum folate levels in Tibetan adults. The relationship between altitude and folate levels should be explored in the future in populations of different races and disease states. Further large-scale prospective studies are needed to illustrate the exact causality of this relationship.

## Supplementary Information

Supplementary Information 1.

Supplementary Figure S1.

## Data Availability

The datasets generated or analyzed during the current study are not publicly available due to ethical restrictions but are available from the corresponding author upon reasonable request.
