# Supplementary material for: The association between altitude and serum folate levels in Tibetan adults on the Tibetan plateau
Source: Sci Rep. 2022 Oct 25;12:17886. doi: 10.1038/s41598-022-22968-6 (PMC9596477; doi:10.1038/s41598-022-22968-6)
Supplement: Supplementary file 1 — Supplementary Information 1. [file 41598_2022_22968_MOESM1_ESM.docx]

# The association between altitude and serum folate levels in Tibetan adults on the Tibetan plateau

Shaoli Yao^1#*^, Xiwen Chen^#2^, Yao Zhou^1^, Li Xu^1^, Qi Zhang^1^, Shimin Bao^1^, Huiru Feng^1^, Weihong Ge^1^

^1^ Department of Neurology, Hospital of Chengdu Office of People's Government of Tibet Autonomous Region, Chengdu, Sichuan, China

^2^ Department of Neurology, The Second Affiliated Hospital of Chengdu Medical College, China National Nuclear Corporation 416 Hospital, Chengdu, Sichuan, China

^#^Shaoli Yao and Xiwen Chen contributed equally to this work

*** Correspondence:**Corresponding Author
E-mail:185540534@qq.com

# Supplementary Table S1

| Table S1 Linear univariate regression analysis of serum folate levels as the dependent variable. | | |
| --- | --- | --- |
| **Variables** | ***β* (95%; CI)** | ***P* value** |
| **Altitude per500 meter** | -0.44 (-0.71; -0.16) | 0.002 |
| **BMI, kg/m^2^** | -0.02 (-0.09; 0.05) | 0.608 |
| **Age, years** | 0.02 (0; 0.04) | 0.099 |
| **MMSE score** | 0.05 (-0.02; 0.11) | 0.158 |
| **HGB, g/L** | -0.01 (-0.03; 0) | 0.015 |
| **RBC, × 10^12^/L** | -0.72 (-1.18; -0.27) | 0.002 |
| **HCT, %** | -0.07 (-0.12; -0.02) | 0.004 |
| **MCV, fL** | 0.01 (-0.02; 0.03) | 0.559 |
| **MCH, pg** | 0 (-0.1; 0.1) | 0.994 |
| **Albumin, g/L** | 0 (-0.07; 0.07) | 0.922 |
| **Glucose, mmol/L** | 0.06 (-0.2; 0.33) | 0.632 |
| **Cholesterol, mmol/L** | 0.24 (-0.07; 0.56) | 0.130 |
| **TG, mmol/L** | 0.24 (-0.26; 0.74) | 0.339 |
| **LDL-C, mmol/L** | 0.31 (-0.08; 0.7) | 0.120 |
| **HDL-C, mmol/L** | 2.67 (1.35; 3.98) | < 0.001 |
| **ALT, U/L** | -0.01 (-0.02; 0.01) | 0.327 |
| **AST, U/L** | 0 (-0.03; 0.02) | 0.811 |
| **Sex** |  |  |
| Male | reference |  |
| Female | 0.68 (0.12; 1.23) | 0.017 |
| **Education** |  | 0.068 |
| Primary or less | reference |  |
| Secondary | 1.23 (0.1; 2.36) | 0.033 |
| Intermediate | 1.03 (0.15; 1.92) | 0.022 |
| University | 0.56 (-0.27; 1.39) | 0.189 |
| **Occupation** |  |  |
| Farmer and herdsman | reference |  |
| Other jobs | 0.5 (-0.71; 1.7) | 0.416 |
| **Smoke** |  |  |
| No | reference |  |
| Yes | -0.52 (-1.16; 0.12) | 0.112 |
| **Alcohol** |  |  |
| No | reference |  |
| Yes | -0.28 (-0.84; 0.28) | 0.331 |
| BMI, Body Mass Index; HGB, Haemoglobin; RBC, Red blood cell; HCT, Hematocrit; MCV, Mean corpuscular volume; MCH, Mean corpuscular hemoglobin; TG, Triglycerides; LDL-C, Low-density lipoprotein cholesterol; HDL-C, High-density lipoprotein cholesterol; AST, Aspartate aminotransferase; ALT, Alanine aminotransferase; MMSE, Mini-Mental State Examination; CI, Confidence interval; | | |

# Supplementary Fig S1

**Fig S1.** Adjusted dose-response association between altitude and serum folate with the RCS function. The model with four knots located at the 5th, 35th, 65th, and 95th percentiles. Y-axis represents the beta to present serum folate for any value of altitude compared to individuals with 3695 meters of altitude. Adjustment factors included age, sex, RBC, HCT, HGB, and HDL-C. The black line and gray area represent the estimated values and their corresponding 95% conﬁdence intervals, respectively. The upper limit of the altitude variable is restricted to 99th. Abbreviation: Ref, reference.
