# Supplementary figures and images for: The association between altitude and serum folate levels in Tibetan adults on the Tibetan plateau
Source: Sci Rep. 2022 Oct 25;12:17886. doi: 10.1038/s41598-022-22968-6 (PMC9596477; doi:10.1038/s41598-022-22968-6)

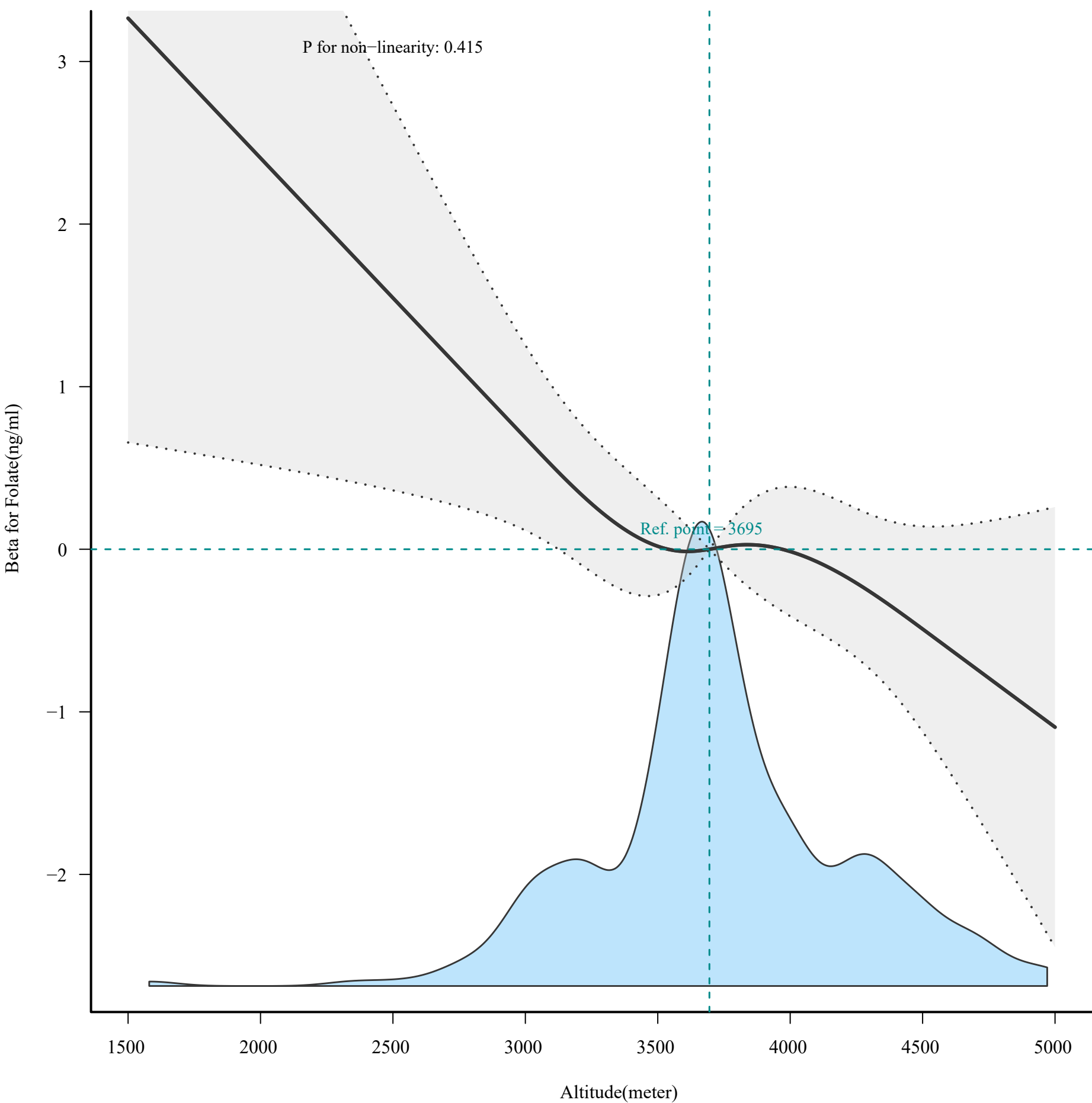

Supplement: Supplementary file 2 — Supplementary Figure S1. [file 41598_2022_22968_MOESM2_ESM.pdf]
